# Serum Homocysteine Could Be Used as a Predictive Marker for Chronic Obstructive Pulmonary Disease: A Meta-Analysis

**DOI:** 10.3389/fpubh.2019.00069

**Published:** 2019-04-04

**Authors:** Deepti Chaudhary, Nidhi Sharma, Sabyasachi Senapati

**Affiliations:** Department of Human Genetics and Molecular Medicine, Central University of Punjab, Bathinda, India

**Keywords:** COPD, homocysteine, folic acid, meta-analysis, systematic review, susceptibility

## Abstract

**Background:** Serum homocysteine (Hcy) level is inversely related with concentration of folic acid, which is an essential micronutrient for metabolism and energy homeostasis. Serum concentrations of Hcy have been reported to have strong correlation with smoking, which is a major risk factor for pathogenesis of chronic obstructive pulmonary disease (COPD) irrespective of ethnicity and gender. Therefore, we performed a systematic review based meta-analysis to evaluate the overall contribution of Hcy in COPD.

**Method:** Published literature on association of serum Hcy with COPD were obtained through conventional web search and eligible literature were selected based on stringent inclusion/exclusion criteria. Continuous variable data was presented as mean and standard deviation. The variable data was analyzed using RevMan 5 statistical tool to meta-analyze mean differences (MD) with 95 % CI for case-control studies.

**Result:** Four case-control studies met the inclusion criteria for this study. A total of 145 COPD subjects and 107 healthy controls were analyzed. Elevated serum homocysteine concentration was found to induce risk for COPD (*MD* = 3.05).

**Conclusion:** Molecular role of Hcy in COPD pathogenesis or prognosis is not clear but existing literature suggests that smoking disturbs folic acid metabolism and promotes Hcy accumulation. This study suggested the contribution of Hcy in COPD pathogenesis. However, large scale prospective cohort study and replication studies with more power are warranted to confirm the results.

## Introduction

Chronic obstructive pulmonary disease (COPD) is an immune-mediated inflammatory disease affecting lung function. It is predicted that by 2020, COPD will be the third most pandemic disease across the globe (1). It contributes considerably to the overall disease burden, morbidity and mortality worldwide (2). It has emerged as critical global health issue, with cigarette smoking being a pivotal risk determinant along with several other factors, such as exposure to indoor and outdoor air pollutants, occupational hazards, and infections (3–6). COPD is more frequent among elderly individuals (age >45 years) and disease burden is predicted to increase with increasing median age of a population (7). Findings have suggested that patients with advanced COPD (GOLD stage III or IV) usually suffer from malnutrition and negative energy balance (8, 9). Recent studies identified an association between intake of micronutrient (including folic acid) deficit food below recommended dietary allowance (RDA) and risk of developing COPD (7).

Folic acid or vitamin B9 is a water-soluble micronutrient that plays a major role in general metabolism and energy homeostasis. COPD is a disease characterized by complex nutritional abnormalities and lower level of vitamin B9 and B12 (10). Folate is essential for the synthesis of nucleic acids and regulation of gene expression epigenetically through one-carbon metabolism (11, 12). Evidences support that folic acid plays central role in metabolism of homocysteine (Hcy). Homocysteine is sulfur containing non-proteinogenic amino acid (isoform of amino acid cysteine), which acts as an intermediate in the metabolism of methionine in the folic acid or folate pathway. Folate helps in homocysteine methylation and thus reduces the risk of cardiovascular diseases (10). As observed among general population, mild to moderate hyperhomocysteinemia (HHcy) is associated with folate deficiency. Methylene tetrahydrofolate reductase (MTHFR) is a key determinant enzyme, which has been shown to be associated with HHcy (13). Homocysteine plays a critical role in the pathogenesis of atherosclerosis and is suggested to be involved in COPD. Serum concentration of Hcy is associated with the severity of COPD (1, 3, 14). Serum Hcy level is influenced by several factors including gender, body mass index (BMI), cigarette smoking, blood pressure (BP). Elevated level of Hcy correlates with poor folic acid status (13, 14). Recent study reported a higher median of total homocysteine (tHcy) value among COPD patients as compared to healthy controls. Independent association of smoking with elevated tHcy among COPD was also reported (1).

At molecular level, there are evidences of systemic inflammation in COPD patients, measured either as increased circulating cytokines such as IL-6, TNF-α, IL1-β, chemokines, and acute phase proteins such as CRP, fibrinogen, serum amyloid A, surfactant protein D, or as abnormalities in circulating cells such as monocytes, neutrophils, lymphocytes and NK cells (15). Chemokines such as CXCL8 (IL8) also play an important role in neutrophil and monocyte recruitment, and elevated circulating CXCL8 concentration is found to be associated with COPD (4). Alpha 1 antitrypsin deficiency, a major biomarker for COPD has been shown to be influenced by environmental exposures, but the molecular cross talk has not been elucidated till date (5).

We hypothesized that elevated serum Hcy provides significant risk to develop COPD. Limited reports are available where association of serum Hcy level was tested with COPD. Objective of our study was to perform first ever meta-analysis to establish the association of serum concentration of Hcy with COPD.

## Materials and Methods

In this study, we have performed a systematic review to evaluate the association of COPD (outcome) with serum concentration of homocysteine (exposure) by performing meta-analysis using data from published literature in the public domain.

### Literature Retrieval

Published literature were searched using NCBI-PubMed, Embase and Google scholar. We included all the relevant literature available till July 2018. To maintain the requisite standard of the study, we included studies which were: (a) available in the public domain; (b) published in peer-reviewed journals; and (c) written in English language. The aim of the literature search was to identify reports where the association between serum homocysteine concentration and onset of COPD has been studied. Following keywords were used for literature search such as: “folic acid and chronic obstructive pulmonary disease”, “folic acid in COPD”, “vitamin B9 in COPD”, “folate and COPD”, and “homocysteine and COPD.” Related cross-reference articles from these retrieved literature were also searched manually to identify additional eligible studies.

### Inclusion and Exclusion Criteria

Literature were included in the present study based on defined inclusion and exclusion criteria. Only cross sectional case-control studies were included where serum concentration of Hcy were measured among COPD patients and healthy controls. We included studies where diagnosis and recruitment of COPD cases were done as per GOLD criteria (16). We did not consider smoking status, gender information or any other anthropometric/physiological features as inclusion/exclusion criteria. Reviews and mere abstracts were excluded from this meta-analysis. We did not restrict our study to a particular population and have included all the relevant published reports.

### Data Extraction

Data was independently extracted by two investigators and resolved differences and contradictions by group discussions. Detailed information such as first author name, publication year, study design, numbers of COPD patients and controls, and the level of serum tHcy in COPD (mean ± standard deviation) were extracted from each of the eligible articles.

### Statistical Analysis

Serum concentration of tHcy was taken as a marker for folic acid metabolism in this meta-analysis. Combined analysis was performed without stratifying the study cohort on the basis of smoking history. All the continuous data (serum tHcy concentration) was presented as mean ± SD. Serum concentration given as median and range were converted into mean and standard deviation (SD) using the formula reported by Wan et al. (17) and Hozo et al. (18).

$$\begin{array}{l} \text{S}.\text{D}=\frac{b-a}{4};\bar{X}=\frac{a+2m+b}{4} \end{array}$$

where, b = upper range, a = lower range, $\bar{X}=$ mean, m = median, and 4 is considered for the 100% accuracy.

For the moderate sized sample set (15< *n* ≤ 70), formula: $\frac{range}{4}$, is the best estimator for the standard deviation (S.D).

Meta-analysis was performed using Review Manager 5.3.5 (RavMan 5.3.5) Copenhagen: The Nordic Cochrane Center, The Cochrane Collaboration, 2014. Mean differences (MD) with 95% confidence interval (95% CI) were plotted for all the eligible studies on a forest plot to estimate the pooled MD. Study heterogeneity was estimated by chi-square *p*-value and *I*^2^. DerSimonian and Laird random effect model was used to perform the meta-analysis. To estimate the study precision and visually detecting the study bias or systematic heterogeneity, funnel plots were observed. Effect estimates (x-axis) were plotted against standard errors (on reverse scale) to identify any study with falling beyond 95% confidence interval.

## Results

### Study Search

**Figure 1:**
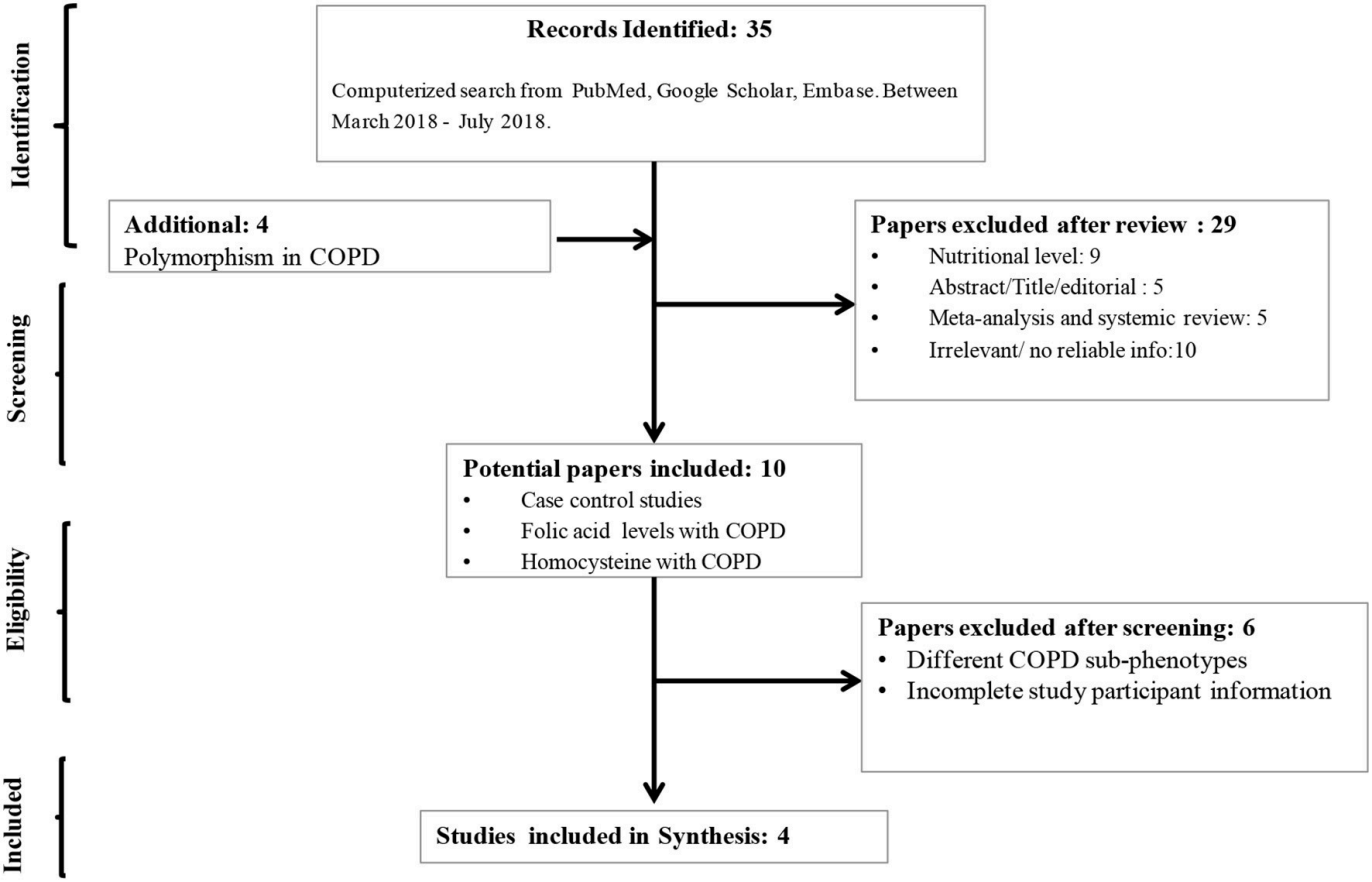
Flow-diagram showing strategy to identify eligible literature for the present study. Three of these studies included both smokers and non-smokers and one study included only smokers.

Based on the mentioned web-search strategies, a total of 35 articles were obtained. After removal of review articles, studies not related with the topic, commentary articles, and others including polymorphisms, 10 studies were identified for further evaluation. In addition, studies lacking essential information or without full text were excluded. Finally, four studies that met the inclusion criteria were included in the present meta-analysis (Supplementary Table 1). A flowchart of the search and selection process is shown in Figure 1.

### Association Between Serum Homocysteine and COPD

**Figure 2:**
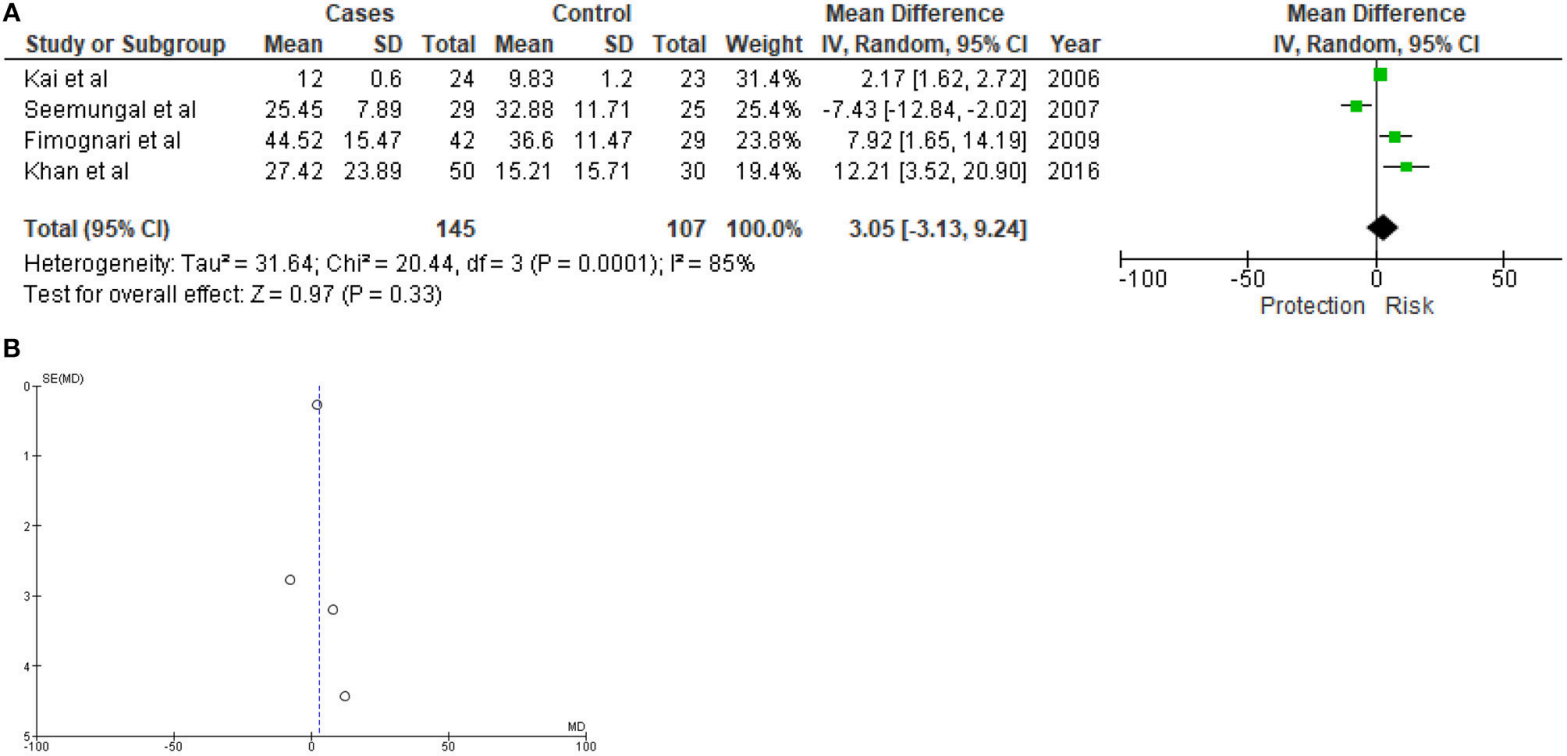
Forest plot **(A)** and funnel plot **(B)** showing results of meta-analysis. A total of 145 COPD subjects and 107 healthy controls were evaluated.

Meta-analysis was performed using DerSimonian and Laird random-effect model due to high degree of data heterogeneity (*I*^2^ = 85%; chi^2^ *p* = 0.0001). Serum concentration of Hcy of 145 COPD patients and 107 healthy controls were evaluated, where 56 COPD patients and 54 healthy controls were non-smokers. Elevated serum homocysteine concentration was identified as risk factor for COPD. However *p*-value of the *Z*-test was not significant (*MD* = 3.05; 95% *CI* = −3.13 to 9.24; *p* = 0.33). Visual inspection of funnel plot indicated no detectable study biasness in this meta-analysis (Figure 2). Sensitivity analysis using Knapp-Hartung method could not be performed as only one study (3) was found to have relevant Hcy concentration data for subgroup analysis.

## Discussion

Very few studies were performed to evaluate the association between Hcy and COPD. Ours is the first systematic review based meta-analysis to investigate this association. Present meta-analysis suggested considerable risk attributed by elevated serum homocysteine in pathogenesis of COPD, however, it did not stand 5% level of significance, which could probably be due to limited number of samples (Figure 2). Due to limited number of studies with smaller sample sets, and lack of subgroup data in eligible literature, overall power of this study was limited and quality assessment analysis was not performed, respectively. Serum concentration of Hcy is a highly inconsistent variable and its metabolism is dependent on several other factors. Therefore, a wide range of confounding factors may potentially impact such study findings. This could also explain the discordant study findings by Seemungal et al. (1) from other three studies included in the meta-analysis. All the four eligible studies included in this meta-analysis were cross sectional studies and did not provide enough statistical data to investigate the effects of known confounding factors on the outcome. Evidences from ecological study or cohort study are not available to support the risk of higher serum Hcy in developing COPD and in the estimation of associated confounding factors, such as smoking, different metabolic disorders/conditions, neuroendocrine, etc.

Presently there is no evidence on molecular impact of reduced serum Hcy concentration and onset of COPD. However, smoking could be directly involved in altering serum Hcy concentration that leads to altered tissue-specific epigenetic signature(s) to promote COPD pathogenesis. In the transmethylation pathway, methionine synthase causes the re-methylation of Hcy to methionine. Folate is one of the co-factor for methionine synthase and requisite for the removal of Hcy by transmethylation. Inadequate folate level creates hindrance in the removal of Hcy and increases its concentration. Thus, high intake of folate is associated with low Hcy level (19, 20). Folate consumption is a deciding factor in case of homocysteine (Hcy) associated thickening of carotid artery, which is ultimately related with the coronary heart diseases (CHD), and stroke. There is an inverse relationship between serum folate level and the risk of CHD (19, 21). According to a retrospective cohort study by Morrison et al. (21), probability of lethal CHDs are more in individuals with low folate level. This study has revealed in the prospective Nutrition Canada Survey that the chances of lethal cardiovascular diseases were related with low folate levels in 165 deaths among 5,056 men and women who were observed for 20 years. This increased risk of cardiovascular diseases is not confined to the individuals with low serum folate levels but was also noticed among individuals having normal serum level indicating that appropriate serum folate level needs to be determined (21).

Significantly lower buccal mucosal folate concentration was reported among current smokers (*n* =39) compared to non-current smokers (*n*=60) in a cross-sectional study carried out by Piyathilake et al. (22). Same study also identified 42% reduction of serum Hcy concentration among smokers as compared to non-smokers if the folate intake is 65μg/d, which is normally a high intake value (3X RDA). This finding indicated inverse relation between smoking and bioavailability of Hcy (22). Another cohort study on 2,435 Dutch men and women identified serum tHcy concentration to be inversely associated with folate intake, where an increase in one quintile folate intake is associated with significantly greater decrease in tHcy among smokers (*P*-trend < 0.001) as compared to non-smokers (*P*-trend < 0.01) (23). This finding highlights that serum tHcy concentration is more sensitive to intake of folate among smokers compared to non-smokers (23). Significantly higher serum homocysteine concentrations among male smokers were observed compared to female smokers in a cohort study comprising 2,282 men and women (24).

In summary, this study highlighted Hcy as a potential risk factor for COPD. Available reports till date either suffer from lack of study power or did not consider known confounding factors to access the outcome. Present study thus suffers from high degree of statistical as well as biological heterogeneity. Stratified prospective cohort study is warranted to access the cause-effect relationship and association between Hcy and COPD.

## Author Contributions

DC and SS conceptualized, designed, and performed the study. SS and DC were involved in data analysis. DC, NS, and SS wrote the manuscript. All the authors reviewed the manuscript before submitting.

### Conflict of Interest Statement

The authors declare that the research was conducted in the absence of any commercial or financial relationships that could be construed as a potential conflict of interest.
