# Supplementary material for: Serum Homocysteine Could Be Used as a Predictive Marker for Chronic Obstructive Pulmonary Disease: A Meta-Analysis
Source: Front Public Health. 2019 Apr 4;7:69. doi: 10.3389/fpubh.2019.00069 (PMC6458233; doi:10.3389/fpubh.2019.00069)
Supplement: Supplementary file 1 [file Data_Sheet_1.docx]

**Supplementary Table 1:** Demographic characteristics of groups included in the meta-analysis.

| **Extracted statistical data** | | | | | | | | |
| --- | --- | --- | --- | --- | --- | --- | --- | --- |
| **PARAMETERS** | **STUDY 1** | | **STUDY 2** | | **STUDY 3** | | **STUDY 4** | |
| Publication (year) | Kai *et al*. (2006) | | Seemungal *et.al.* (2007) | | Fimognari*et.al*. (2008) | | Khan *et.al*. (2016) | |
|  | Cases | Controls | Cases | Controls | Cases | Controls | Cases | Controls |
| No. of individuals | 24 | 23 | 29 | 25 | 42 | 29 | 50 | 30 |
| Age | 70.7±1.3 | 66.4± 2.60 | 69.1 | 64.8 | 71.38+_8 | 70.6+_5.8 | 58.3+_9.2 | 51.5+_7.6 |
| Smokers | 1 | 7 | 8 | 1 | 42 | 29 | 38 | 16 |
| Non-Smokers | 23 | 16 | 21 | 24 | -- | -- | 12 | 14 |
| BMI | 20.7 ± 0.7 | 24.2±0.8 | 24.0 | 27 | 26.5+_4.4 | 28.1+_4.1 | 21.36+_4.2 | 22.8+_4.6 |
